# Prevalence of Consistent Condom Use and Associated Factors among Serodiscordant Couples in Ethiopia, 2020: A Mixed-Method Study

**DOI:** 10.1155/2021/9923012

**Published:** 2021-09-30

**Authors:** Wolde Melese Ayele, Tesfaye Birhane Tegegne, Yitayish Damtie, Muluken Genetu Chanie, Asnakew Molla Mekonen

**Affiliations:** ^1^Department of Epidemiology & Biostatistics, School of Public Health, Wollo University, Ethiopia; ^2^Department of Reproductive and Family Health, School of Public Health, Wollo University, Ethiopia; ^3^School of Public Health, Wollo University, Ethiopia

## Abstract

**Background:**

Heterosexual transmission within serodiscordant relationships is the core source of new HIV infections. Although consistent condom use can significantly reduce HIV transmission risk among serodiscordant couples, it has not been extensively studied in Ethiopia. Consequently, the current study looked at the proportion of serodiscordant couples in Ethiopia who used condoms consistently and the factors associated with that.

**Methods:**

A cross-sectional was conducted from October 2019 to June 2020. For the quantitative findings to be more robust and reliable, a qualitative design was incorporated. An interviewer-administered questionnaire was used to collect the data. Qualitative data were collected using gender-matched four focus group discussions. Multivariable logistic regression was conducted to identify factors associated with consistent condom use. The statistical significance of the variables was declared at a *P* value of less than 0.05.

**Results:**

This study confirmed that the proportion of consistent condom use was 58.4% [95% CI: 53.1-63.1%]. After controlling for all other variables, unmarried partners, adjusted odds ratio (AOR) = 0.44 [95% CI: 0.229-0.877] and students and employees, AOR = 0.33 [95% CI: 0.130-0.846] and AOR = 0.39 [95% CI: 0.165-0.939], respectively, were less likely consistently use condoms, whereas couples living together, AOR = 1.86 [95% CI: 1.197-2.195], receiving counseling about condom use, AOR = 1.90 [95% CI: 1.182-3.076], and having more knowledge about HIV, AOR = 1.61 [95% CI: 1.031-2.525] were more likely to use condoms consistently.

**Conclusion:**

Despite its importance, the proportion of consistent condom use among serodiscordant couples was significantly low. To improve condom use consistently, planners, policymakers, and health care practitioners should consider the factors listed above when making decisions. There should be an increased focus on student and employee intervention as well.

## 1. Introduction

Human immunodeficiency virus (HIV) continues to be a significant public health issue. The disease has claimed more than 35 million lives globally [1].

Sub-Saharan Africa is the most affected region that accounts for about two-thirds of the global total new HIV infections. The vast majority of people living with HIV (PLWHA) are from low-and middle-income countries including Ethiopia [2].

The majority of new infections of HIV are through the heterosexual transmission of HIV among the serodiscordants [3, 4]. Repeated low-risk sexual acts will be translated into a significant cumulative risk over time, especially with unprotected sexual experiences [5].

In sub-Saharan Africa, it is estimated that half of the HIV-positive people have a negative couple. The percentage of serodiscordant partnerships is 0–6% in generalized epidemics and 9–17% in concentrated epidemics of HIV [6, 7]. As per the findings of previous studies, the risk of transmission among serodiscordant couples is predicted by many variables. These include sociodemographic [8–10], personal behavior [8, 11–13], cultural [14, 15], and lack of awareness [16].

Abstaining from sex, having sex with only one uninfected partner, using condoms are well-known ABC methods of preventing HIV transmission [17]. Among those prevention methods, consistent and correct condom use is a critical component in a comprehensive and sustainable approach to the prevention of HIV [2, 18–22]. The world has exceeded the AIDS targets of the Millennium Development Goal (MDG) 6, halting and reversing the spread of HIV. Currently, many countries are getting on the fast-track targets to end the AIDS epidemic by 2030 as part of the Sustainable Development Goals [1, 23].

Despite its significance, consistent condom use is underutilized [24]. The problem is dominant among the serodiscordant couples. The issue is significantly high in sub-Saharan Africa, including Ethiopia [25, 26]. Among HIV serodiscordant couples with HIV, 65-85% have acquired the infection from their partners [27, 28]. Deprived of any interventions, the annual rate of HIV transmission is 6.3% among the negative serodiscordant partners [29].

According to the previous studies, low utilizations of consistent condom use have been associated with different factors. Cost of condom, religious ideology, alcohol or drug use, younger sexual debut, poor knowledge of HIV/AIDS, beliefs of diminished sexual pleasure if used condom and male emotional fulfillment, disbelief in prevention efficacy, trust in relationships, gender inequality and perceptions of modesty, partner characteristics and type of relationship, lower education and unemployment, and psychological problems were among the independent predictors [30–33].

There is a global target of achieving the elimination of new HIV infections by 2030. Controlling the continuing threat of new HIV infection is one of the core health priorities of the Federal Ministry of Health of Ethiopia. Hence, this study is aimed at contributing its part. The result will also ensure the continuum of care so that healthy couples and their offspring will be transformed into healthy adolescents. Although we need such healthy people, HIV serodiscordant couples are increasing through time. This is a source of new infection for seronegative spouses and newborn babies. Therefore, this study is aimed at finding the magnitude and factors associated with consistent condom use. The findings will have a crucial input to the policymakers and planners in updating and designing interventions in reducing new HIV infections. Moreover, identifying the predictors of consistent condom use will be important in developing an effective sensitization program for couples and enabling them to be informed and knowledge-based partner relationship creation. The result will also have vital importance for clinicians, public health specialists, nongovernmental initiatives, and future researchers in HIV among couples.

## 2. Material and Methods

### 2.1. Study Area and Period

This study was conducted from October 2019 to June 2020 in Dessie city public health institutions. Dessie is the main city of the South Wollo Zone, Amhara Regional State. It is located 401 kilometers to the north of Addis Ababa, the capital of Ethiopia. The Zone has an estimated population of 3,034,327, of which Dessie city comprises 218,471(unpublished observation, Zonal Annual Plan) total population. In the city, there is one comprehensive specialized hospital and four health centers giving ART services. All the ART providing institutions were included in the study.

### 2.2. Study Design and Study Participants

A facility-based cross-sectional study was conducted. For the findings of inconsistent use of condoms more robust and reliable, a mixed-method study was conducted among the serodiscordant couples. The study participants were all HIV-positive spouses among the discordant couples who had ART follow-up. The recruited participants were HIV-positive partners of the serodiscordant couples, sexually active during the study period, at least 18 years of age, aware of their HIV serological status, and could provide informed consent to participate in the study. Participants were excluded if they were single or had separated from their sexual partners. In addition, participants who were unable to communicate during the data collection period were excluded from the study. Similarly, four gender-matched foci group discussions (FGDs) were conducted. The participants for the qualitative study were the seropositive couples who were on ART follow-up. Two FGDs were employed in each gender that used condoms consistently and inconsistently.

### 2.3. Sample Size Determination and Data Collection Procedure

The sample size was computed using a single population proportion formula by considering the following assumptions: proportion of consistent condom use (55.8%) in Northwest Ethiopia [34], the 95% confidence level, a 5% margin of error, and 10% nonresponse rate.

(1) $$\begin{array}{c} n=\frac{\left({\left(Z\alpha /2\right)}^{2}\right)\left(p\left(1-p\right)\right)}{d^{2}}=379\; \end{array}$$

where *n* is the minimum sample size, *Z* is the critical value of the desired level of confidence 95% (1.96), *P* is the proportion of consistent condom use in a study of Northwest Ethiopia, and *d* is the margin of error (5%).

Finally, it yields a total of 417 samples after adding 10% nonresponse rates. The number of participants in the qualitative design was determined based on the information gained to attain the objectives stated. Therefore, a total of 36 (17 females and 19 males) participated from both consistent condom users and nonusers. We did not use any criteria to recruit the participants except gender and consistent condom use status in the FGD.

A structured interviewer-administered questionnaire was designed based on the existing previous studies. The tool was initially developed in English, translated to Amharic, and then pretested to ensure its cultural acceptance and understanding by the study subjects and data collectors. The data were collected at their point of exit during their ART follow-up period. HIV-positive and ARV-treated individuals who are in an intimate relationship with HIV-negative individuals are considered a discordant couple. Consistent condom user of discordant couples was defined as if the heterosexual couples used condoms during every act of sexual contact without interruption throughout their sex life until the data collection period.

Semistructured guide questions were used during the FGD. The data were collected using text writing by a reporter and video recording to maximize the consistency during synthesis and transcription. The discussion was led by the investigators of this research project.

### 2.4. Data Processing and Analysis

Epi Data version 3.1 and STATA version 14 statistical package softwares were used for data entry and analysis, respectively. Data were coded according to the nature of the variables. The descriptive statistics were used to compute the frequency distribution of categorical variables and means and standard deviations (SD) for continuous variables. A multivariable logistic regression model was constructed to identify an independent association between consistent condom use and associated factors and to control the potential confounding variables. The Hosmer and Lemeshow test was applied to test the logistic regression model fitting. In bivariate analysis, independent variables with *P* values less than or equal to 0.2 were recruited for the multivariate logistic regression model. In multivariable regression, variables with a *P* value of less than 0.05 were considered statistically significant. Adjusted odds ratio (AOR) with 95% confidence Interval (CI) was computed to determine the direction and strength of the statistical association. Similarly, qualitative data were analyzed by theme based on the nature of the issues raised by the group members.

### 2.5. Ethical Considerations

The study was approved by the Ethical Review Committee (ERC) of the College of Medicine and Health Sciences, Wollo University. The letter of permission to conduct the study was obtained from the respective administrative offices of study sites. Likewise, signed consent was obtained from each study participant. Information about the purpose and benefit of the study was provided to the participants. They were informed that participating in the study was voluntary-based. The right to withdraw from the study at any moment during the interview was guaranteed. No personal identifiers were used on the data collection form. The recorded data was not accessed by a third person except the investigators and was kept confidential and anonymous. This study did not conduct experiments involving human subjects. All the procedures of the study were conducted according to the Helsinki Declaration of ethical approval and consent to participate.

## 3. Results

### 3.1. Characteristics of Participants

A total of 401 serodiscordant couples were participated in this study, making a 96.2% response rate. The respondents' age ranged from 18 to 72 years, with a mean (±SD) of 34.9 ± 10.86 years. Two hundred and nineteen (54.6%) of the respondents were female partners. More than two-thirds of 401 participants were urban dwellers, 289 (72.1%). Regarding the couple's monthly income, a large proportion of the couples, 217 (54.1%), reported a monthly household income below the median 2000 Ethiopian Birr. The sociodemographic descriptive table is shown in Table 1.

### 3.2. Participants of the FGD

Four focus group discussions were held among individuals in seropositive couples. Group arrangements were designed according to gender. Two FGDs were comprised of female seropositive, while the rest were males. The group formation was HIV seropositive female (g1*n*1 = 9), HIV seropositive female (g2*n*2 = 8), HIV seropositive males (g3*n*3 = 7), and HIV seropositive males (g4*n*4 = 12). Group one (g1) and group three (g3) from each gender were from the serodiscordant couples who used condoms consistently, while the other groups were inconsistent condom users.

The mean age of males was 36 while of females was 29 years. Twenty and eight females and 12 males in each group reported having had sex with someone besides their stable partner in the past twelve months. Six and three females had their own jobs that can survive them independently, while all males had jobs that can earn money for them monthly.

### 3.3. Behavioral and Knowledge Variables

In this study, most participants lived together with their partners, 226 (56.4%). Among the pairings who participated in the study, 109 (27.2%) were temporarily paired. In the last 12 months of this study, seventy-seven (19.2%) of the participants had sexual intercourse with someone other than their partners. Widely held, 299 (74.6%) of the seropositive partners have used a condom during their last sexual intercourse. Among couples who used condoms, 188 (50.2%) were decided by both partners. During each antiretroviral therapy (ART) follow-up, only 229 (57.1%) HIV-positive couples received condom use counseling from a healthcare provider. Similarly, 231 (57.6%) partners denied sexual intercourse whenever their seronegative partners refused to use condoms. The overall prevalence of consistent condom use among couples with discordant serum was 58.4% [95% CI: 53.1-63.1%]. Behavioral and knowledge variables are presented in Table 2.

### 3.4. Factors Associated with Consistent Condom Use

After adjusting for confounding variables, in multivariable regression analysis, marital status, occupation, couples living together, knowledge, and received counseling about condom use were the independent predictors of consistent condom use among the serodiscordant couples.

Unmarried seropositive partners were 56% less likely to use condoms consistently compared to married serodiscordant couples, AOR = 0.44 [95% CI: 0.229-0.877].

Likewise, compared to housewives, the probability of consistent condom use as a partner of a student and an employee is reduced by almost 70% and 60% with AOR = 0.33 [95% CI: 0.130-0.846] and AOR = 0.39 [95% CI: 0.165-0. 949], respectively.

Spouses who lived together were twice more likely to use condoms consistently compared with those who did not live together with their spouses AOR =1.86[95% CI: 1.197-2.195].

In terms of counseling, couples who received counseling about condom use during ART appointment were more likely to use condoms consistently than their counterparts, AOR = 1.90 [95% CI: 1.182-3.076].

Similarly, compared to unaware couples, the rate of consistent condom use upon intercourse with a partner was nearly doubled among knowledgeable partners, AOR = 1.61 [95% CI: 1.031-2.525]. The overall regression results are shown in Table 3.

### 3.5. Factors Explored by FGD

The majority of the FGD members (10 females and 12 males) reported a high level of openness with their spouses about their sexual needs. Fewer females than males said that they trusted their spouses.

More females than males were happy with the decision to use condoms in every sexual intercourse.

Based on the FGD result, persuasion, refusing sex without condoms, and crying were the methods that females used to use condoms consistently.

Consistent condom user females persuade their seronegative partners by using different methods. Of which, they express their heartfelt love for their male partners. They told that their partners agreed to use a condom during this situation. Similarly, if females were economically independent, they refused sexual intercourse without a condom.

“I used different methods to decide in using condoms consistently. Sometimes, I tried to persuade my partner as I could not use other pregnancy prevention methods. Other times, I cried to him as he did not love me. Moreover, he insults me. During that time, I just shouted out”(g1, p3).

Almost all of the females who did not use a condom consistently replayed that not because of their problem. They reported that they were very interested in whether they always used condoms, but they have been used inconsistently for different reasons. Among that, their economic dependency on their male partners was a very crucial and novel factor. Although females are seronegative, they were enforced not to use a condom unless their male partners need it. This is because females rely on their partners' income to support themselves.

“g2, p5: I have been living with my sexually active partner for six years. We knew his seropositivity after two years of our start of living together. I was seronegative during his serological test time. We are using condoms inconsistently. We also got a child after we knew his HIV status. It was because of the need for my partner alone. I refused, but he insulted me aggressively. I do not have relatives. Also, I do not have a job to earn money. Therefore, I am obligated to live with him by using condoms inconsistently. By now, I do not know my HIV status. It was because of my economic dependency” (she masked her face using a cloth to hide her crying).

Among the inconsistent condom users, two females did not disclose their HIV status to their male partners. They uttered that they were economically dependent on their partners. Therefore, if disclosed, they will be distressed, and their partners will either dispatch or kill them.

“I know that it is a sin that I closed my seropositivity. But I do not have any choice. If I disclosed to my partner, I am sure that he would chase me without any property. Sometimes, we used a condom. He asked me the reason why I tried to use condoms. I replied that I had gastritis to use an oral contraceptive, and I am sick to take other family planning methods. Sometimes, he trusted me, and we used condoms.” (Crying) (g2, p10).

Regarding males, five used condoms consistently in love. Males' initiation on condom use did not upset their female partners. For some partners, discussing condom use went smoothly, and negative expectations were solved by discussion.

“Using condoms for us is a pleasure. We have been advising about the importance of condoms in my ART follow-up. Sometimes, my partner brings a condom as a surprise. I also receive it because there is nothing more than a condom for our life. I will never forget that one day she said to me:

“Inkoklish: to mean (take a puzzle)”,

I replied “man yawkilsh: to mean (who would know yours?)”,

“Which is not a bar of gold, or not money, etc., and nobody will ever give you it as a gift, but only I brought it. What is that?”

I tried to think more but could not answer it. According to Ethiopian culture, I gave a country like Addis Ababa, Bahir Dar, and so for but she refused to receive it. Instead, she asked me to give her something myself. I agreed,

“That is a “condom”, she laughed. I was very surprised. Therefore, I advise everyone to use condoms consistently. If they need to have a child, they shall consult their doctors” (g3 p23).

On the other hand, males who did not use condoms consistently had different reasons. Six of twelve did not disclose their HIV status to their partners. Three were using condoms consistently with their stable partner but had other casual sexual intercourse without condoms. The other three believed that they accepted any health condition that would occur on their partners. They want to live together and to pass away also.

“I believe that HIV is a payment given by God for our sin. However, I am ready to share everything that comes with my wife. I knew that she was HIV positive three years earlier. But we did not use condoms even once since we knew her HIV status. I tested myself three times, and the last was seven months ago. Although I am ready to share the burden, my HIV test result is negative. Of course, my wife always nags me to use a condom. But I refused and told her the spiritual reality of our relationship” (g4, p32).

“I did not remember the occasion when I was infected with HIV. But now I am HIV-positive fifteen years back. I remember my wife was seronegative after three months of my positivity. I did not know her HIV status now. We used condoms inconsistently. Sometimes, she cried that she was in gastric pain and complained because of the oral contraceptive. During that time, we tried to use a condom. Otherwise, we did not use it. I do not desire to use it, and using condoms had too less sexual pleasure” (g4, p29).

“Even I have a doubt; some people believe that a condom contains a chemical that makes males impotence. But I did not accept it fully. As you defined, a person who did not use condoms for the sake of a child is an inconsistent user. Sometimes, we did not use condoms. For example, we got two children after we knew my HIV seropositivity.” (g4, p33). “By the way, let me add one thing, there are also people who believed condom contains HIV, which is the source for many people infection”.

The other findings mentioned by the FGD had similar themes with the quantitative findings of this study.

## 4. Discussion

Despite various and modernized interventions used, HIV continues to cause a catastrophic effect on human health. Among those interventions, consistent and correct condom use has paramount help to reduce HIV transmission among the serodiscordant partners. The maximum protective effect of condoms reaches if the partners use condoms consistently. On the contrary, if there is inconsistent use of a condom by people living with HIV (PLWHIV), it will worsen HIV infection, reinfection with new drug-resistant viral strains, and newborn acquisition. This study provided crucial findings of the prevalence and determinants of consistent condom use among heterosexual serodiscordant couples in Ethiopia.

The current study revealed that the prevalence of consistent condom use among serodiscordant partners was 58.4% (95% CI: 53.1-63.1%). Despite its need, the proportion of regular condom use in this study was not satisfactory. The main reasons that the serodiscordant couples reported for not using condoms consistently were they desire to have a child 38 (9.5%), their feeling that condom use reduces sexual pleasure 88 (21.9%), and no need to use with no reason 55 (13.7%). This finding is consistent with the studies in Northwestern Ethiopia (55.8%) [35] and Mekelle (55.7%) [36]. This agreement of the findings might be since the sociodemographic characteristics of the participants, the study design applied, and sample size similarities of the studies. Similarly, it is congruent with the research finding in Kenya (57.4%) [33] and Portugal (53%) [37].

The proportion of consistent condom use in this study is higher when compared to the studies in Nigeria (45.8%) [38] (29.4%) [39]. This difference might be due to the study time in which the latter was conducted seven years ago. Currently, many interventions are being implemented to increase the consistent use of condoms. These might have an implication to increase the prevalence of consistent condom use in the current study.

However, the result is lower than the findings from the studies done in Gondar, Ethiopia (78.9%) [40] Guatemala (85.0%) [41], and Cambodia (78%) [42]. This discrepancy might be due to the lack of professional's commitment to creating awareness on PLWHIV on ART to use a condom consistently. In this study, nearly half of 172 (42.7%) serodiscordant couples have not received advice about condom use. Similarly, receiving advice during ART follow-up was a statistically significant variable to increase consistent condom use. It implies that health professionals should improve advising the clients.

After adjusting the potential confounders in multivariable regression marital status, occupation, partners living together, receiving advice about condom use, and knowledge of participants were the independent predictors of consistent condom use.

This study revealed that unmarried couples were roughly 60% less likely to use condoms consistently compared to married couples. This finding is supported by the conclusion of the researchers in Gondar [43] and Portugal [37].

Unlike previous studies [40, 43, 44] that have reported participants with higher educational status were more likely to use condoms consistently compared to lower educational status participants, this study did not show any significant association between educational status and consistent condom use. Although being urban [43] and rural [40] residents were more likely to use condoms consistently, this study discovered that residency of participants' did not show a statistically significant association with consistent condom use. This variation might be due to the study setting, sample size, and methodological difference of the studies.

Compared to housewives, students and employed participants were less likely to use condoms consistently. In this study, students were the least in monthly income compared to other occupation categories. This fact might be a reason for being unable to buy condoms for every act of sexual intercourse. In addition, students were in the fire age group in this study. These might be the attributes of inconsistent use of condoms. Likewise, employees have a higher probability of contacting different and many people compared to housewives. This situation might increase to have multiple sexual partners. Out of those participants who had multiple sexual partners, 35 (45.5%) did not use a condom consistently. This veracity might be the reason for the employees not using condoms consistently.

Consistent condom use was statistically associated with couples living together. Consistent condom users were more likely to have been living together compared with inconsistent users. This incongruity might be because if partners are not living together, they will have another sexual partner that might favor them to use condoms inconsistently. However, this finding is contradicted with a multilevel analysis study [45] and a preprint finding. The discrepancy might be due to the study population in which the previous study was conducted among drug users.

Comparatively, participants who had received condom advice were more likely to use a condom consistently than their counterparts. This finding is in line with a study in Gondar [40].

This study provides information about the presence of a statistical association between the knowledge of the participants and consistent condom use. Those serodiscordant couples who have good knowledge were as much as use condoms consistently. Demonstration and education about condom use can increase knowledge about the benefit of a condom. This intern improves consistent condom use [45, 46]. Similarly, access to information such as through media and health institutions may contribute to increasing general knowledge about the importance of consistent condom use [46]. Hence, this intervention might increase consistent condom use among discordant couples.

The present findings have several implications in preventing new HIV infection from serodiscordant couples. Unmarried couples should be the focus of HIV prevention strategies and programs if we want to eliminate new HIV infections by 2030.

A school and employer-based HIV prevention program that focuses primarily on the dissemination of information about condom use will be critical in promoting students and employees to use condoms consistently.

Prevention programs should also use a variety of interactive health education and communication strategies, such as advice at every ART follow-up visit and use of different media and workplaces, to be the most effective.

Furthermore, this study suggests that the ministry of labor and social affairs strengthens and promotes serodiscordant couples to live together as a result of the findings. Laws and regulations should be in place to make this requirement effective.

## 5. Conclusions

Contrary to its importance, the percentage of couples who consistently used condoms was low. Independent predictors of consistent condom use among serodiscordant couples were unmarried couples, students, employees, couples who live together, advice received, and participant knowledge. Planning officials and healthcare professionals should consider the factors listed above to improve consistent condom use. The qualitative design examined variables such as female economic dependence, the belief that condom use could cause male impotence, the belief that condom itself contain HIV, and arrogance in religious beliefs. Because of these, future researchers are encouraged to quantify those variables, which are used to alter or develop new HIV prevention strategies. Defensively, this study did not look at the new HIV incidence or the importance of preexposure/postexposure prophylaxis among serodiscordant couples, which should be assessed in future studies. Preexposure prophylaxis, in particular, is not investigated in Ethiopia.

## Figures and Tables

**Table 1:** Sociodemographic characteristics of participants.

Characteristics	Frequency	Percent
Age of participants (mean ± SD), 34.9 ± 10.86		
18-24	76	19.0
25-35	151	37.7
36-48	127	31.7
49 and above	47	11.7
Gender of the participants		
Male	182	45.4
Female	219	54.6
Participants residency		
Rural	112	27.9
Urban	289	72.1
Religion		
Orthodox	130	32.4
Muslim	210	52.4
Protestant	51	12.7
Others	10	2.5
Participants' occupation		
Housewife	55	13.7
Student	71	17.7
Employee	102	25.4
Others	173	43.1
Educational status of participants		
Unable to read and write	81	20.2
Able to read and write	85	21.2
Grades 1-8	29	7.2
Grades 9-12	102	25.4
Diploma and above	104	25.9
Numbers of alive children		
None	114	28.4
1-5	248	61.8
Above 5	39	9.7
Average monthly income of spouses in Ethiopian Birr (median = 2000)		
Median and below	217	54.1
Above the median	184	45.9

**Table 2:** Consistence condom use, behavior, and knowledge-related characteristics of participants, 2020.

Characteristics	Frequency	Percent
Condom use		
Consistence	234	58.4
Inconsistence	167	41.6
Partners live together		
Yes	226	56.4
No	175	43.6
Couples type		
Temporary	109	27.2
Permanent	292	72.8
Years you live together after know seropositive		
<3 years	188	46.9
≥3 years	213	53.1
Make sexual intercourse with other than your partner in the past 12 months?		
Yes	77	19.2
No	324	80.8
Gave birth after know seropositivity		
Yes	176	43.9
No	225	56.1
Did you use condom in your last sexual intercourse?		
Yes	299	74.6
No	102	25.4
Who decided to use condom? (*N* = 377)		
Respondent	68	18.2
Partner	118	31.6
Both partners	188	50.2
Take medication for sexually transmitted diseases in last six months*?*		
Yes	77	19.2
No	324	80.8
Did you use medication or alcohol to increase sense of intercourse?		
Yes	117	29.2
No	284	70.8
Used family planning methods other than condom?		
Yes	140	34.9
No	261	65.1
Took training about condom use?		
Yes	188	46.9
No	213	53.1
Received advice about condom use in every visit?		
Yes	229	57.1
No	172	42.9
Knowledge variables		
Could HIV transmit to you partner if not use condom?		
Yes	270	67.3
No	131	32.7
Condom can prevent HIV transmission during sexual intercourse?		
Yes	260	64.8
No	141	35.2
Did you denied sexual intercourse if your partner do not need to use condom?		
Yes	231	57.6
No	170	42.4
Your partner can survive for long time by protecting from HIV by using condom?		
Yes	268	66.8
No	133	33.2
Antiretroviral treatment can protect HIV for partner?		
Yes	223	55.6
No	178	44.4

**Table 3:** Bivariable and multivariable regression of factors associated with consistence condom use among serodiscordant couples, 2020.

Characteristics	Consistent condom use		COR (95% CI)	AOR (95% CI)	*P* value
	Yes	No			
Marital status					
Married	139	89	1	1	
Unmarried	35	28	0.80 (0.455, 1.460)	**0.44 (0.229, 0.877)**	**0.001**
Partner	40	21	1.21 (1.675, 2.203)	0.91 (0.395,2.127)	0.61
Others^a^	20	29	0.44 (0.233, 0.828)	0.69 (0.277,1.754)	0.10
Seropositive partner occupation					
Housewife	29	26	1	1	
Students	48	23	1.87 (0.905, 3.867)	**0.33 (0.130, 0.846)**	**0.01**
Employee	65	37	1.57 (0.809, 3.064)	**0.39 (0.165, 0.939)**	**0.03**
Others^b^	92	81	1.01 (0.555, 1.870)	0.93 (0.458, 1.874)	0.83
Educational status					
Unable to read and write	49	32	0.77 (0.424 ,1, 419)	0.73 (0.327, 1.653)	0.53
Able to read and write	44	41	0.54 (0.302, 0.980)	1.01 (0.461, 2.209)	0.15
Levels 1-8	21	8	1.33 (0.535, 3.308)	0.53 (0.189, 1.513)	0.55
Levels 9-12	51	51	0.50 (0.289, 0.890)	1.83 (0.884, 3.796)	0.33
College and above	69	35	1	1	
Residency					
Rural	61	51	0.80 (0.516, 1.145)	1.18 (0.708, 1.984)	0.54
Urban	173	116	1	1	
Monthly average income of the spouses in Ethiopian Birr (median = 2000)					
Median and below	136	81	1	1	
Above the median	98	86	0.72(0.485,0.1.070)	0.80(0.486,1.335)	0.26
Did you live together with your partner?					
Yes	119	107	1	1	
No	115	60	1.72(1.147,2.589)	**1.86(1.197,2.195)**	**0.009**
Type of partner					
Temporary	69	40	0.58(0.36,0.937)	0.67(0.383,1.937)	0.47
Permanent	218	74	1	1	
Did you use medication/alcohol before sexual intercourse?					
Yes	72	45	1	1	
No	162	122	0.82(0.534,1.289)	0.85(0.514,1.419)	0.83
Received advice about condom use at every ART visit					
Yes	173	56	1.57(1.015,2.432)	**1.90(1.182,3.076)**	**0.04**
No	114	58	1		
Knowledge of participants about condom					
Knowledgeable	163	100	1.53(1.014,2.332)	**1.61(1.031,2.525)**	**0.03**
Not knowledgeable	71	67	1	1	

^a^Widowed, separated, and divorced; ^b^daily laborers, farmer, merchant, pension. The bold confidence intervals and *P* values indicate the presence of statistical significance of variables.

## Data Availability

All the necessary data are included in the manuscript.

## Abbreviations

AOR: Adjusted odds ratio
FGD: Focus group discussion
g1: Group 1
g2: Group 2
g3: Group 3
g4: Group 4
OR: Odds ratio
*p*: Person in each group.
